# Amyloid Precursor Protein (APP) and GABAergic Neurotransmission

**DOI:** 10.3390/cells8060550

**Published:** 2019-06-06

**Authors:** Bor Luen Tang

**Affiliations:** 1 Department of Biochemistry, Yong Loo Lin School of Medicine, National University of Singapore, Singapore 117597, Singapore; bchtbl@nus.edu.sg; Tel.: +65-6516-1040; 2 NUS Graduate School for Integrative Sciences and Engineering, National University of Singapore, Singapore 117597, Singapore

**Keywords:** amyloid precursor protein (APP), amyloid-beta (Aβ), gamma-aminobutyric acid (GABA), GABA receptor, potassium chloride cotransporter 2 (KCC2)

## Abstract

The amyloid precursor protein (APP) is the parent polypeptide from which amyloid-beta (Aβ) peptides, key etiological agents of Alzheimer’s disease (AD), are generated by sequential proteolytic processing involving β- and γ-secretases. APP mutations underlie familial, early-onset AD, and the involvement of APP in AD pathology has been extensively studied. However, APP has important physiological roles in the mammalian brain, particularly its modulation of synaptic functions and neuronal survival. Recent works have now shown that APP could directly modulate γ-aminobutyric acid (GABA) neurotransmission in two broad ways. Firstly, APP is shown to interact with and modulate the levels and activity of the neuron-specific Potassium-Chloride (K^+^-Cl^−^) cotransporter KCC2/SLC12A5. The latter is key to the maintenance of neuronal chloride (Cl^−^) levels and the GABA reversal potential (E_GABA_), and is therefore important for postsynaptic GABAergic inhibition through the ionotropic GABA_A_ receptors. Secondly, APP binds to the sushi domain of metabotropic GABA_B_ receptor 1a (GABA_B_R1a). In this regard, APP complexes and is co-transported with GABA_B_ receptor dimers bearing GABA_B_R1a to the axonal presynaptic plasma membrane. On the other hand, secreted (s)APP generated by secretase cleavages could act as a GABA_B_R1a-binding ligand that modulates presynaptic vesicle release. The discovery of these novel roles and activities of APP in GABAergic neurotransmission underlies the physiological importance of APP in postnatal brain function.

## 1. Introduction

Alzheimer’s disease (AD) [1] is the most prevalent cause for aging-associated dementia [2]. The amyloid cascade hypothesis [3] posits that the accumulation and deposition of the amyloid-beta (Aβ) peptides in the brain parenchyma is a crucial step in disease development [4]. Aβ peptides are generated from the amyloid precursor protein (APP) through sequential cleavages by the β-secretase Beta-site APP Cleaving Enzyme 1 (BACE1) and the Presenilin-containing γ-secretase complex [5]. However, a first APP cleavage by the α-secretase ADAM10 [6] would effectively preclude Aβ formation. Much of the AD research over the years has focused on attempts to better understand the BACE1-γ-secretase-mediated amyloidogenic pathway, as well as searching for means to inhibit APP proteolysis or to decrease amyloid load. Although it is now clear that proteolytic processing of APP is complex [6,7,8,9] and no clinical trial of anti-Aβ drugs have shown any clear benefits to date [10], Aβ remains a prime AD therapeutic target [11,12] and continues to garner research efforts and interests.

APP is itself known to have a range of activities in the brain that are indicative of its physiological importance [13,14,15]. Mammals have three paralogous genes which encode APP and two APP-like proteins (APLP1 and APLP2) [16]. Although APP knockout in mice produce viable and fertile offspring, APP-deficient adult mice exhibit decreased locomotor activity compared to wild-type, as well as signs of neuroinflammation [17]. Various combinations of genetic deficiencies of the three members of the APP family resulted in early postnatal death and neurodevelopmental defects [18,19], attesting to both overlapping as well as non-redundant functions of the APP paralogues. Although fairly ubiquitous in its expression, a good number of physiological roles for APP and its non-amyloid cleavage products are known to affect neurons and neurotransmission. These include neurite/axon outgrowth [20,21,22], axonal guidance [23], neural cell adhesion [24,25], neuronal survival [26,27,28], and neural progenitor cell-fate determination [29,30]. Most importantly, APP is involved in the modulation of synaptic neurotransmission and plasticity. Both pre- and post-synaptic protein compositions are altered in neurons bearing APP mutant transgenes [31], or those in APP knockout [32] mice. The changes include reductions in the key postsynaptic neurotransmission components Postsynaptic density protein 95 (PSD-95) and the α-amino-3-hydroxy-5-methyl-4-isoxazolepropionic acid (AMPA) subunit GluR1. APP is a synaptic adhesion molecule [25,33,34] and has both presynaptic [35,36] and postsynaptic [37] localization and functions [38]. Aβ is well known for causing pathological dysregulation of postsynaptic trafficking of both the AMPA [39] and *N*-Methyl-d-aspartate (NMDA) [40]-type glutamate receptors. Notably, APP also has physiological roles in the function and trafficking of these glutamate receptors [41,42,43] and may thus be important for synaptic plasticity and learning/memory [44,45,46,47,48]. The actions of APP at the synapse are also known to be mediated by the secreted (s)APPs, mainly sAPPα generated by α-secretase cleavage [49,50,51,52,53].

γ-aminobutyric acid (GABA), a major inhibitory neurotransmitter in the brain, shapes brain tissue activity and provides a balancing stability to neural systems and networks [54] by preventing uncontrolled hyper-excitation (such as those occurring during epileptic episodes [55]). GABAergic neurotransmission is mediated by the ionotropic GABA_A_ receptors (GABA_A_R) [56], as well as the metabotropic GABA_B_ receptors (GABA_B_R) [57]. GABA_A_R functions as ligand-gated chloride (Cl^−^) channels and whether GABA binding would be depolarizing or hyperpolarizing is largely determined by intracellular Cl^−^ concentrations and the GABA reversal potential (E_GABA_). Resting Cl^−^ concentration in central nervous system (CNS) neurons is determined by the activity of two major cation-chloride cotransporters, namely the Cl^−^ influx-mediating Na^+^-K^+^-2Cl^−^ cotransporter 1 (NKCC1) and the efflux-mediating K^+^-Cl^−^ cotransporter 2 (KCC2) [58]. In the adult brain, GABA is mainly hyperpolarizing and inhibitory, but it is primarily depolarizing and excitatory in developing neurons, as demonstrated using rat embryonic and neonatal cortical slices [59]. This is largely because embryonic or immature neurons have high levels of NKCC1 but low levels of KCC2. However, KCC2 expression is developmentally upregulated in mature neurons, resulting in an increase in intracellular Cl^−^, with GABA thus becoming hyperpolarizing and inhibitory [60]. Changes in KCC2 expression and activity may thus underlie neuropathological conditions [61,62,63] associated with weakened GABA signaling due to a positive shift in E_GABA_.

Other than modulating the activity of excitatory glutamate receptors, recent works have now shown that APP could also directly modulate GABA neurotransmission via its interaction with KCC2 and its alteration of intracellular Cl^−^ [64,65]. Furthermore, APP or its soluble cleavage product could interact with GABA_B_R to modulate presynaptic GABA_B_R-mediated inhibition or presynaptic vesicle release [66,67]. In the paragraphs that follow, an update of these findings is provided and the new perspectives brought about by these findings are discussed.

## 2. Amyloid Precursor Protein (APP) and Gamma-Aminobutyric Acid (GABA)ergic Neurotransmission

There are some earlier indications that APP modulates GABAergic transmission. In the loss-of-function context of the APP knockout mouse, an impairment in synaptic plasticity, as demonstrated by deficiencies in Long-term potentiation (LTP) formation [68,69] and behavioral/learning deficits [70], is associated with a reduction in GABA-elicited inhibitory post-synaptic currents [69]. Also, theta-gamma oscillation phase-amplitude coupling involving inhibitory transmission was strongly diminished in recordings from the parietal cortex and hippocampus of APP knockout mice [71]. APP is highly expressed in the GABAergic neurons in the neurogenic dentate gyrus, and selective deletion of APP in GABAergic, but not glutamatergic neurons disrupted adult hippocampal neurogenesis [72]. In this regard, it is notable that the excitatory activity of GABA on newborn neurons at the dentate gyrus is critical for synapse formation and dendritic development [73] and APP would thus play a role in GABA transmission for newborn neurons in embryonic neonatal as well as adult neurogenic settings. APP also appears to interact with and regulate the levels of Ca(v)1.2, the channel pore subunit of L-type calcium channels downstream of depolarizing GABA neurotransmission in neurons of the striatum and hippocampus. Changes in GABAergic short-term plasticity in these neurons with the loss of APP may therefore be related to this interaction [74]. Taken together, perturbations in GABAergic inhibitory transmission in CNS neurons resulting from the loss of APP attested to the latter’s function in modulating the former.

Some findings in the context of APP over-expression are also in support of its role in GABAergic neurotransmission. Controllable transgenic over-expression of APP in transgenic mice from birth (but not over-expression in adults) resulted in epileptiform electroencephalogram abnormalities which are not related to Aβ levels or plaque load, and are unaffected by a γ-secretase inhibitor [75]. In a mouse model of Down syndrome (DS), with mice harboring an extra chromosome 16 on which APP is located, GABA_A_R signaling was in fact found to be excitatory rather than inhibitory in hippocampal slices from the DS mice [76]. This appears to be associated with an increase in hippocampal NKCC1 expression and an inhibition of NKCC1 activity was able to reverse the phenotype. Taken as a whole, APP over-expression appears to have the effect of altering GABAergic neurotransmission by shifting the neuronal GABA reversal potential. In the section below, new findings on how APP influences this shift are discussed.

## 3. APP’s Modulation of GABAergic Neurotransmission through Potassium Chloride Cotransporter 2 (KCC2)

In investigating changes in NKCC1 and KCC2 levels and GABA responses in rat cortical neurons in culture, Doshina et al. [65] noted an increase in KCC2 and a decrease in NKCC1 levels with increasing days in vitro (DIV). These changes corresponded with a reduction in the neurons’ GABA depolarizing potential beginning 7 DIV, and which became greatly reduced by 13–17 days in vitro (DIV). Adenoviral vector-mediated over-expression of human APP in these neurons decreased both the transcript and protein levels of KCC2. However, unlike previous findings in the chromosome 16 trisomy mice [76], NKCC1 levels were unaffected by APP over-expression. Downregulation of KCC2 by APP elicited a more depolarizing GABA response, as indicated by an increase in intracellular Ca^2+^ due to signaling downstream from GABA_A_R [77] in late DIV neurons. However, unlike previous observations made with APP knockout mice [74], there were no significant changes in the levels of Ca(v)1.2. The notion that APP-induced changes in GABA response are due mainly to changes in intracellular Cl^−^ resulting from KCC2 downregulation is supported by its reversal by the NKCC1 inhibitor bumetanide. Importantly, the authors showed some in vivo relevance of their findings in culture neurons by showing that Adenovirus-associated virus (AAV) construct-based transduction of APP in brains of mouse pups also reduced KCC2 levels without affecting NKCC1.

How does over-expressed APP downregulate KCC2? This APP activity is independent of the APP intracellular domain (AICD) (which is known for its transcriptional activities [78]), APP’s extracellular domain, or γ-secretase cleavage. However, APP over-expression is correlated with a decrease in the expression of upstream stimulating factor 1 (USF1), a known transcriptional regulator of the KCC2-encoding *SLC12A5* gene [79]. Although it is unclear at the moment how APP affects the expression of USF1, the findings indicate that it is an important factor in maintaining KCC2 levels, intracellular Cl^−^, and E_GABA_ in adult brain neurons.

In another report, Chen et al. [64] noted a depolarizing shift of E_GABA_ in hippocampal slices of APP knockout mouse. By patching a glutamatergic neuron in a hippocampal culture and recording for post-synaptic unitary inhibitory postsynaptic current (uIPSC) of neighboring GABAergic interneurons, the mean uIPSC amplitude is found to be significantly reduced in APP knockout neurons compared to wild-type. Interestingly, analysis of hippocampal tissue lysates revealed a significant and specific reduction in the levels of the α1-subunit of GABA_A_R (which mediates fast inhibition). As with Doshina et al. [65], Chen et al. also noted a reduction in total and plasma membrane KCC2 levels (but not NKCC1) in an APP-deficient hippocampus. Both KCC2 levels and function could in fact be restored pharmacologically by Cl^−^ extrusion enhancers such as CLP257 and CLP290 [80]. Importantly, restoration of normal KCC2 expression and function in APP-deficient mice with the CLPs reversed the changes in E_GABA_ and GABA_A_R α1 levels as well as GABA_A_R mediated inhibition. The changes observed in APP-deficient neurons could thus be largely attributed to the reduction of KCC2 levels and activity, although it is yet unclear why GABA_A_R α1 levels were specifically reduced in the absence of APP.

On the other hand, Chen et al. [64] elucidated a different mechanism for APP deficiency-induced reduction in KCC2. The authors showed with co-transfection experiments that full-length APP, but not its proteolytic fragments, stabilized KCC2 levels. Functional expression of KCC2 at the neuronal cell surface is necessary for its Cl^−^ efflux activity, and the trafficking of KCC2 to the cell surface and its subsequent endocytic internalization is regulated by different cellular mechanisms, with defects in these known to underlie a range of neuropathological conditions [58]. One such regulatory mechanism is the tyrosine phosphorylation of KCC2 mediated by tyrosine kinases, such as Src [81,82,83], which promotes KCC2 internalization from the plasma membrane and its subsequent lysosomal degradation. Interestingly, Chen et al. found that APP and KCC2 interacts physically by co-immunoprecipitation and proximity ligation assays. Moreover, levels of KCC2 tyrosine phosphorylation are increased in the absence of APP, correlating with its lower levels, and this is effectively reduced by a Src family tyrosine kinase inhibitor. It appears that APP’s interaction with KCC2 may limit its tyrosine phosphorylation, thus maintaining the former’s expression and activity at the plasma membrane. Increased tyrosine phosphorylation, however, is not the only reason why KCC2 is reduced in APP-deficient cells, as the levels of non-phosphorylatable mutants of KCC2 (Y903A and Y1087A) are still low in cells not co-expressing APP. Notably, the levels of ubiquitinated KCC2 in an APP-deficient hippocampus are significantly increased compared to wild-type, and the proteasome inhibitor MG132 increased levels of the mutant KCC2 only in the absence but not in the presence of the co-expressed APP. APP–KCC2 interactions thus appear to also limit KCC2 ubiquitination.

The findings of the two reports discussed above indicated that APP could be a physiological regulator of KCC2 expression and function, which would be consequently critical for neuronal intracellular Cl^−^ concentrations and inhibitory neurotransmission. It appears that APP could regulate E_GABA_ by modulating KCC2 levels in different ways, both influencing the latter’s transcript level through a major transcription factor as well as enhancing KCC2′s plasma membrane stability through limiting its susceptibility to post-translational modifications in the form of tyrosine phosphorylation and ubiquitination.

## 4. APP’s Modulation of Presynaptic GABA_B_ Receptor (GABA_B_R) Activity

Presynaptic glutamate and GABA receptors modulate neurotransmitter release [84] and the action of presynaptic GABA_B_R in this regard has been well-documented [85,86,87]. The two subtypes of GABA_B_R, namely GABA_B_R1 and GABA_B_R2, typically form functional heterodimers. There are two isoforms of GABA_B_R1, GABA_B_R1a and GABA_B_R1b, which differ by the presence of two N-terminal sushi domain repeats that are unique to GABA_B_R1a [88]. These sushi repeats confer differential plasma membrane domain targeting of GABA_B_Rs. While GABA_B_R1b-containing GABA_B_Rs are targeted dendritically and mediate postsynaptic inhibition, GABA_B_R1a-containing GABA_B_R are axonal and inhibit glutamate release from the presynaptic plasma membrane [88]. The sushi repeats appear to aid axonal targeting in this regard [89,90]. Proteomics analyses have shown that GABA_B_R1a/GABA_B_R2 receptors co-purify with the kinesin-1 motor adapters, like the c-Jun N-terminal kinase-interacting protein (JIP) and Calsyntenin [91], attesting to the notion that these are trafficked to axons via kinesin-1-mediated axonal transport. However, as the sushi domains of GABA_B_R1a are extracellular/luminal, they need to be linked to the cytoplasmic kinesin-1 by yet-to-be-identified transmembrane domain-containing proteins. A high-resolution proteomics screen has in fact identified some potential sushi domain interacting membrane proteins, including APP [91].

Dinamarca et al. [66] have now further investigated APP, as well as two other proteins that bind with high affinity to the sushi domains of GABA_B_R1a and form distinct complexes with GABA_B_R. These molecules are of interest as they could potentially function in linking GABA_B_R1a to the axonal-targeting motor protein-adaptor complex. Among these sushi domain interactors, only the loss of APP impaired GABA_B_R-mediated presynaptic inhibition. In this regard, the GABA_B_R agonist baclofen was less able to reduce the amplitude of the evoked excitatory postsynaptic current (EPSC), as well as the frequency of miniature EPSC, in APP-deficient compared to wild-type hippocampal slices. APP was previously known to associate with both JIP [92] and calsyntenin [93], and confirmation of the interactions in this regard attested to APP’s potential to function as a transmembrane linker that facilitates axonal transport of GABA_B_R. Interestingly, complex formation with GABA_B_Rs stabilizes APP at the cell surface and appears to reduce amyloidogenic processing of APP to Aβ. Thus, other than APP serving a GABA_B_R axonal transport role to the presynaptic plasma membrane, the APP-GABA_B_R complex formation may potentially also influence APP proteolysis and Aβ formation.

If full-length APP could interact with GABA_B_R1a, sAPPs which encompass APP’s ectodomain, might be able to do likewise. A recent proteomics screen by Rice et al. [67] has indeed uncovered that sAPPα’s extension domain (ExD) [94] binds directly to the sushi 1 domain of GABA_B_R1a. This sAPPα–GABABR1a interaction reduced the release probability of synaptic vesicles and suppressed synaptic transmission. This inhibition of synaptic vesicle release underlies sAPPα’s apparent enhancement of short-term plasticity at Schaffer collateral synapses of hippocampal slices in a GABA_B_R1a-dependent manner. In fact, a 17–amino acid peptide within sAPPα’s ExD was able to replace sAPPα’s activity in this regard, and when infused into the hippocampal region suppressed in vivo spontaneous neuronal activity of CA1 pyramidal cells in mice. These findings indicate that GABA_B_R1a act as a high-affinity synaptic receptor for sAPPα, mediating a physiological role for sAPPs in modulating synaptic transmission.

## 5. New Perspectives

**Figure 1:**
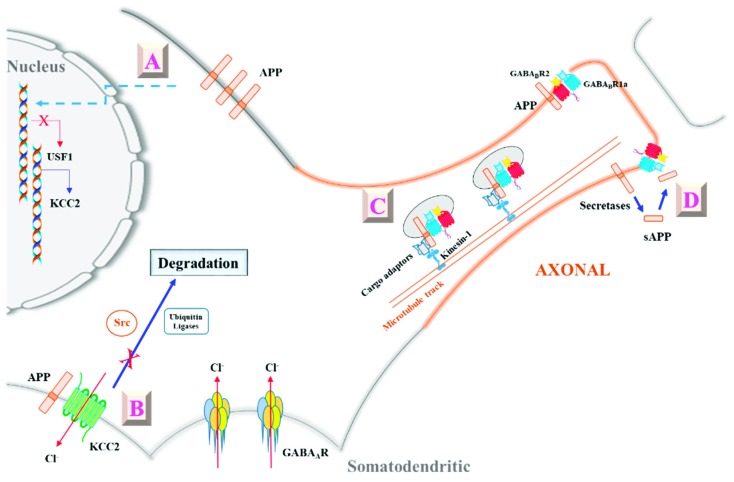
A schematic diagram illustrating amyloid precursor protein’s (APP’s) modulation of γ-aminobutyric acid (GABA)ergic neurotransmission via its interaction with potassium chloride cotransporter 2 (KCC2) and GABA_B_ receptor 1a (GABA_B_R1a). A: APP modulates KCC2 expression by suppressing the levels of a KCC2 transcription factor UCF-1 in an unknown manner [65]. B: APP can also interact directly with KCC2, and maintains its levels and stability by inhibiting tyrosine phosphorylation and ubiquitination-based degradation [64]. APP’s modulation of KCC2 levels alters intracellular Cl^−^ and shifts E_GABA_, thereby affecting inhibitory signaling through GABA_A_Rs. C: APP’s interaction with GABA_B_R1a’s sushi domain allows it to effectively aid axon targeting of GABA_B_R1a-GABA_B_R2 dimers [66]. D: Furthermore, secreted (s)APP generated by secretase cleavage could bind as a ligand to GABA_B_R1a to modulate GABA_B_R’s presynaptic roles [67]. See text for more details.

The findings described above (summarized in Figure 1) provided some fresh perspectives on APP’s role in GABAergic neurotransmission. That APP could modulate KCC2 levels (and thus intracellular Cl^−^ levels) either at the transcriptional or post-translational level would mean that the former has an important role to play in inhibitory neurotransmission, not just through the ionotropic GABA_A_R but also through the glycine receptor [95], another ligand-gated chloride channel. APP could thus moderate neuropathological conditions by maintaining intracellular Cl^−^ levels and attenuate depolarizing shifts in E_GABA_, which would heighten excitation-based neuropathology. However, the actual in vivo relevance of APP’s function in this regard remains to be determined. Future work shall reveal whether there are changes in APP levels under neuropathological conditions that might lead to changes in KCC2 levels and intracellular Cl^−^, or APP polymorphisms that might predispose an individual to conditions like epileptic seizures or neuropathic pain. From a cellular and molecular perspective, much remains to be learned with regard to the physical and functional interactions between APP and KCC2. As alluded to above, how APP deficiency affects KCC2 transcription is not yet fully deciphered. Furthermore, the nature and dynamics of the APP–KCC2 interaction that effectively reduces KCC2 access by tyrosine kinases and ubiquitin ligases remains unclear, and whether this interaction has, in turn, any bearings on APP’s proteolytic processing is also not known.

APP’s high-affinity interaction with the sushi domain of GABA_B_R1a gives rise to two new functional perspectives pertaining to metabotropic GABAergic signaling, particularly at the axonal presynaptic compartment where GABA_B_R1a-GABA_B_R2 dimers are selectively targeted to. The basis of this selective axonal targeting could now be, at least partly, attributed to GABA_B_R1a’s interaction with APP as the latter is primarily an axonal protein by way of its engagement of the axonal trafficking kinesin-1 and the motor adaptors JIP and Calsyntenin. This presynaptic targeting of GABA_B_R is important for the latter’s modulation of excitatory neurotransmitter release in hippocampal neurons [96]. Intriguingly, APP proteolytic products containing the APP ectodomain harboring the sushi domain interacting motif (the ExD) could also bind GABA_B_R1a at the presynaptic compartment. sAPP, acting as an agonistic GABA_B_R ligand, thus provides an added dimension to the regulation of GABA_B_R activity at the presynaptic compartment. Again, the extent to which this sAPP-based modulation occurs in vivo is not yet clear. Presumably though, sAPP’s effect on postsynaptic GABA_B_R signaling would be minimal, as these would largely consist of GABA_B_R1b-GABA_B_R2 which lack the sushi domain [88]. Like APP’s modulation of KCC2 levels and stability, the gross effect of APP’s action on GABA_B_R appears therefore to be one that limits excitatory neurotransmission.

There are two neurological implications associated with the findings discussed above. The first pertains to the role of APP and its proteolytic products in synaptic plasticity. In this regard, it is notable that (1) APP proteolytic processing is enhanced by synaptic activity [97,98] and (2) GABA_B_R is an important mediator of homeostatic synaptic plasticity [96]. As pointed out by Rice et al., the authors’ observation “...raised the possibility that the sAPP-GABABR1a interaction acts as an activity-dependent negative-feedback mechanism to suppress synaptic release and maintain proper homeostatic control of neural circuits” [67]. That APP and its cleavage product have key roles in homeostatic plasticity is very much in line with the abundance of APP at the synapse [99], as well as the synaptic deficits associated with loss of APP in mice [68,69], the latter of which could be at least partially rescued by sAPPα [44,100]. GABA_B_R has also been implicated in several neurological disorders [101,102] and is a recognized therapeutic target in this regard [103]. The APP-interacting sushi domain could thus be a drug target that is unique to presynaptic GABA_B_R1a.

The second neurological implication concerns APP’s role in AD pathology, beyond that of being a source of Aβ. APP has been shown to act as a cellular receptor for Aβ [104,105,106] and is known to mediate the pathological effects of Aβ and tau [107,108] in AD models. On the other hand, sAPPα’s neuronal pro-survivor activity is well known [26,28] and in this regard appears to be antagonistic to the neurotoxic nature of Aβ. APP’s role in AD pathology could thus be rather context-dependent. Interestingly, GABA_B_R antagonists can improve memory and enhance cognition [109], and have some demonstrated benefits in animal models and patients with mild cognitive impairment (MCI) [110]. Given that hyper-excitability, interneuron dysfunctions, and network abnormalities are features often associated with, and could precede full clinical onset of, AD [111], APP and sAPPα are therefore potentially useful in countering MCI and certain aspects of AD pathology, as demonstrated recently in a mouse AD model [52]. Furthermore, as α-secretase cleavage and sAPPα generation effectively exclude BACE1 processing, therapeutic strategies that enhance α-secretase processing have been proposed to be beneficial to AD in terms of a lowering of amyloid load and enhancing neuroprotection [112,113]. These possibilities, however, remain to be more fully explored. Conversely, APP’s interaction with KCC2 and GABA_B_R1a at the plasma membrane might influence proteolytic processing of the former in a manner that is AD-relevant. While a reduction of proteolysis of APP to Aβ is shown to result from its interaction with GABA_B_R1a [66], the situation is less clear for KCC2. These are all points that might be worth pursuing from a therapeutics perspective.

The growing appreciation of APP’s activity in modulating both the excitatory and inhibitory neurotransmission suggests that it has fundamental, non-pathological roles in the development, maintenance, and functioning of the mammalian CNS. This fresh perspective would guide future investigations and may even help to innovate disease intervention strategies.
